# Supplementary material for: Fetal Bovine Serum Supplementation Enhances Functional Consistency of IGRA Results in Bovine Tuberculosis Diagnostics
Source: Animals (Basel). 2025 Sep 2;15(17):2580. doi: 10.3390/ani15172580 (PMC12427214; doi:10.3390/ani15172580)
Supplement: Supplementary file 1 [file animals-15-02580-s001.zip › Supplementary Table S1.pdf]

Supplementary Table S1. Individual IFN-γ Responses to Bovis and Mitogen Stimulations in 91 Cattle Under Different FBS Conditions, with Classification of Immune Recovery Patterns.

Sample ID	Bovis OD (Day 0)	Mitogen OD (Day 0)	Bovis OD (FBS X)	Mitogen OD (FBS X)	Bovis OD (FBS O)	Mitogen OD (FBS O)	Day 0 Bovis Result	FBS X Bovis Result	FBS O Bovis Result	Bovis Recovery	Mitogen Recovery	Dual Recovery
1	0.00	2.83	0.00	0.22	0.00	0.73	-	-	-	↓	↑	X
2	0.08	2.96	0.03	0.73	0.10	1.56	-	-	-	↑	↑	O
3	0.01	1.08	0.00	0.26	0.00	0.07	-	-	-	↓	↓	X
4	0.10	2.10	0.00	0.37	0.01	0.83	+	-	-	↑	↑	O
5	0.07	2.43	0.03	0.67	0.08	1.35	-	-	-	↑	↑	O
6	0.03	2.03	0.01	0.25	0.06	0.72	-	-	-	↑	↑	O
7	1.30	3.51	0.18	0.95	0.54	1.47	+	+	+	↑	↑	O
8	0.01	0.98	0.00	0.15	0.05	1.25	-	-	-	↑	↑	O
9	0.07	2.09	0.00	0.20	0.00	0.35	-	-	-	↑	↑	O
10	0.04	2.39	-0.02	0.07	-0.01	0.61	-	-	-	↑	↑	O
11	0.01	1.02	0.01	1.48	0.00	0.63	-	-	-	↓	↓	X
12	0.26	3.39	0.01	1.69	0.16	3.15	+	-	+	↑	↑	O
13	0.11	3.40	0.02	2.34	0.02	2.22	+	-	-	↓	↓	X
14	1.41	3.30	0.07	0.17	0.21	0.80	+	-	+	↑	↑	O
15	0.13	3.41	0.07	1.18	0.02	0.48	+	-	-	↓	↓	X
16	0.16	3.43	0.01	0.61	0.02	0.71	+	-	-	↑	↑	O
17	0.04	3.08	0.01	0.67	0.01	0.59	-	-	-	↑	↓	X
18	0.02	3.58	0.01	1.32	0.01	1.10	-	-	-	↑	↓	X
19	0.13	3.58	0.04	2.96	0.11	3.19	+	-	+	↑	↑	O

20	0.30	3.57	0.09	2.81	0.05	2.83	+	-	-	↓	↑	X
21	0.66	3.43	0.07	2.28	0.03	1.72	+	-	-	↓	↓	X
22	0.59	3.52	0.07	1.43	0.06	1.33	+	-	-	↓	↓	X
23	0.01	1.90	0.01	0.03	0.00	0.13	-	-	-	↓	↑	X
24	-0.02	2.27	0.00	0.02	0.00	0.02	-	-	-	↓	↑	X
25	0.21	3.10	0.08	1.70	0.20	1.99	+	-	+	↑	↑	O
26	0.02	2.51	0.01	0.78	0.00	0.30	-	-	-	↓	↓	X
27	0.54	3.26	0.17	0.75	0.12	0.88	+	+	+	↓	↑	X
28	0.31	3.48	0.06	2.06	0.08	2.31	+	-	-	↑	↑	O
29	1.83	3.41	0.30	1.33	0.28	1.92	+	+	+	↓	↑	X
30	0.00	3.63	0.00	2.98	0.00	3.15	-	-	-	↓	↑	X
31	0.05	1.22	0.00	0.05	0.00	0.10	-	-	-	↓	↑	X
32	1.26	3.33	0.45	1.19	0.26	1.86	+	+	+	↓	↑	X
33	0.14	2.68	0.02	0.05	0.01	0.04	+	-	-	↓	↓	X
34	0.01	3.60	0.00	2.00	0.00	1.94	-	-	-	↓	↓	X
35	0.42	3.32	0.13	1.88	0.20	2.94	+	+	+	↑	↑	O
36	0.29	3.07	0.01	0.51	0.23	1.26	+	-	+	↑	↑	O
37	0.02	3.46	0.00	1.23	0.02	1.32	-	-	-	↑	↑	O
38	-0.01	3.32	-0.01	1.17	0.00	1.74	-	-	-	↑	↑	O
39	-0.01	3.66	0.00	1.35	0.01	0.87	-	-	-	↑	↓	X
40	0.38	3.69	0.01	1.97	0.02	2.34	+	-	-	↑	↑	O
41	-0.03	2.14	0.00	0.32	0.00	0.68	-	-	-	↑	↑	O

42	0.13	3.55	0.05	2.49	0.11	2.97	+	-	+	↑	↑	O
43	0.08	3.24	0.01	0.24	0.08	3.15	-	-	-	↑	↑	O
44	1.85	3.48	0.14	1.11	0.66	2.88	+	+	+	↑	↑	O
45	0.01	3.52	0.00	1.55	0.00	0.78	-	-	-	↓	↓	X
46	0.19	3.41	0.00	0.18	0.00	0.42	+	-	-	↓	↑	X
47	0.00	3.23	0.00	0.57	0.00	1.07	-	-	-	↓	↑	X
48	0.05	3.51	0.01	2.82	0.03	1.43	-	-	-	↑	↓	X
49	1.51	3.46	0.17	0.31	0.41	1.40	+	+	+	↑	↑	O
50	2.32	3.62	0.64	1.32	0.97	2.78	+	+	+	↑	↑	O
51	0.10	3.56	0.02	2.35	0.02	2.66	+	-	-	↓	↑	X
52	1.88	3.05	0.05	0.15	0.05	0.39	+	-	-	↑	↑	O
53	0.08	3.40	0.10	3.07	0.05	2.62	-	-	-	↓	↓	X
54	0.03	3.18	0.04	1.46	0.01	1.06	-	-	-	↓	↓	X
55	0.23	3.35	0.02	1.23	0.06	1.09	+	-	-	↑	↓	X
56	0.10	3.61	0.05	2.99	0.03	2.78	+	-	-	↓	↓	X
57	0.21	3.32	0.02	0.17	0.01	0.30	+	-	-	↓	↑	X
58	0.35	3.50	0.02	0.82	0.01	1.77	+	-	-	↓	↑	X
59	0.21	3.24	0.06	0.57	0.16	1.24	+	-	+	↑	↑	O
60	0.10	3.23	0.01	0.81	0.02	1.48	-	-	-	↑	↑	O
61	0.33	3.52	0.07	1.36	0.15	2.54	+	-	+	↑	↑	O
62	0.22	3.43	0.06	0.73	0.23	2.52	+	-	+	↑	↑	O
63	0.31	3.44	0.05	1.69	0.08	2.01	+	-	-	↑	↑	O

64	0.14	3.41	0.00	0.19	0.01	1.42	+	-	-	↑	↑	O
65	1.35	3.41	2.53	2.86	2.93	3.33	+	+	+	↑	↑	O
66	0.00	2.59	0.01	0.67	0.00	1.21	-	-	-	↓	↑	X
67	0.17	3.16	0.03	0.56	0.04	1.38	+	-	-	↑	↑	O
68	1.31	3.46	0.90	2.44	1.43	3.09	+	+	+	↑	↑	O
69	0.06	3.22	0.01	0.18	0.01	0.89	-	-	-	↑	↑	O
70	0.03	3.60	0.00	2.62	0.02	3.02	-	-	-	↑	↑	O
71	0.21	3.56	0.19	2.72	0.30	2.74	+	+	+	↑	↑	O
72	1.12	3.24	0.24	0.93	0.69	2.75	+	+	+	↑	↑	O
73	0.31	3.53	0.05	1.27	0.14	2.26	+	-	+	↑	↑	O
74	0.00	3.40	-0.01	1.73	0.00	2.33	-	-	-	↑	↑	O
75	0.30	3.06	0.02	0.21	0.12	1.65	+	-	+	↑	↑	O
76	0.00	3.33	0.00	1.39	0.00	1.52	-	-	-	↑	↑	O
77	0.07	3.43	0.00	0.59	0.00	1.48	-	-	-	↓	↑	X
78	1.12	3.78	0.13	1.93	0.22	3.13	+	+	+	↑	↑	O
79	0.45	3.39	0.01	0.41	0.19	2.70	+	-	+	↑	↑	O
80	0.06	3.52	0.02	2.10	0.01	0.68	-	-	-	↓	↓	X
81	0.24	3.16	-0.01	0.57	0.02	0.75	+	-	-	↑	↑	O
82	0.41	3.21	0.01	0.53	0.13	1.30	+	-	+	↑	↑	O
83	0.00	2.43	-0.01	0.90	0.00	1.51	-	-	-	↑	↑	O
84	0.00	3.38	0.00	1.57	0.00	2.47	-	-	-	↓	↑	X
85	0.08	3.01	0.02	1.98	0.03	3.01	-	-	-	↑	↑	O

86	0.54	3.04	0.03	0.15	0.06	0.40	+	-	-	↑	↑	O
87	0.16	3.11	0.03	1.38	0.08	1.87	+	-	-	↑	↑	O
88	0.01	3.23	0.00	0.77	0.00	0.20	-	-	-	↑	↓	X
89	0.01	3.22	0.00	0.73	0.00	0.40	-	-	-	↑	↓	X
90	0.00	2.03	0.00	0.31	0.00	0.20	-	-	-	↑	↓	X
91	0.16	3.44	0.03	2.83	0.04	3.11	+	-	-	↑	↑	O

Abbreviations: +, IGRA-positive (OD ≥ 0.1); –, IGRA-negative; ↑, recovery (OD increase compared to FBS X); ↓, decline; O, both Bovis and Mitogen recovered (Dual Recovery); X, not recovered.
