# Supplementary material for: Fetal Bovine Serum Supplementation Enhances Functional Consistency of IGRA Results in Bovine Tuberculosis Diagnostics
Source: Animals (Basel). 2025 Sep 2;15(17):2580. doi: 10.3390/ani15172580 (PMC12427214; doi:10.3390/ani15172580)
Supplement: Supplementary file 1 [file animals-15-02580-s001.zip › Supplementary Table S2.pdf]

Supplementary Table S2. Diagnostic performance of IGRA under different storage conditions (N = 91).

Comparison	Sensitivity (Se)	Specificity (Sp)	PPV	NPV
FBS X vs Day 0	26 %	100 %	100 %	60.2 %
FBS O vs Day 0	52.0 %	100 %	100 %	70.4 %
